# A prospective pilot study using metabolomics discloses specific fatty acid, catecholamine and tryptophan metabolic pathways as possible predictors for a negative outcome after severe trauma

**DOI:** 10.1186/s13049-019-0631-5

**Published:** 2019-05-22

**Authors:** Luis Servià, Mariona Jové, Joaquim Sol, Reinald Pamplona, Mariona Badia, Neus Montserrat, Manuel Portero-Otin, Javier Trujillano

**Affiliations:** 1 Critical Care Unit, University Hospital Arnau de Vilanova, 25198 Lleida, Spain; 2 Department of Experimental Medicine, University of Lleida-Lleida Biomedical Research Institute (IRBLleida), 25198 Lleida, Spain

**Keywords:** Metabolome, Mortality, Traumatic brain injury, Biomarker, Multiple traumatism

## Abstract

**Background:**

We wanted to define metabolomic patterns in plasma to predict a negative outcome in severe trauma patients.

**Methods:**

A prospective pilot study was designed to evaluate plasma metabolomic patterns, established by liquid chromatography coupled to mass spectrometry, in patients allocated to an intensive care unit (in the University Hospital Arnau de Vilanova, Lleida, Spain) in the first hours after a severe trauma (*n* = 48). Univariate and multivariate statistics were employed to establish potential predictors of mortality.

**Results:**

Plasma of patients non surviving to trauma (*n* = 5) exhibited a discriminating metabolomic pattern, involving basically metabolites belonging to fatty acid and catecholamine synthesis as well as tryptophan degradation pathways. Thus, concentration of several metabolites exhibited an area under the receiver operating curve (ROC) higher than 0.84, including 3-indolelactic acid, hydroxyisovaleric acid, phenylethanolamine, cortisol, epinephrine and myristic acid. Multivariate binary regression logistic revealed that patients with higher myristic acid concentrations had a non-survival odds ratio of 2.1 (CI 95% 1.1–3.9).

**Conclusions:**

Specific fatty acids, catecholamine synthesis and tryptophan degradation pathways could be implicated in a negative outcome after trauma. The metabolomic study of severe trauma patients could be helpful for biomarker proposal.

**Electronic supplementary material:**

The online version of this article (10.1186/s13049-019-0631-5) contains supplementary material, which is available to authorized users.

## Background

Severe trauma injuries are the first cause of mortality among population younger than 44. With a global mortality higher than 5 million individuals/year its clinical assessment and care is a first order public health question [1]. The use of clinical scores such as APACHE II and SAPS in intensive care units (ICU) have improved the follow-up [2]. However, even considering those scores specifically developed for trauma injuries (e.g. TRISS, TRAM, RISC), these exhibit a large space for improvement in calibration and discriminative power [3].

Accordingly, there is a need for biomarker characterization in order to improve precise measurements of severity and pathophysiological consequences, hereby improving their early, scientifically-guided, therapeutic approaches overall leading to a better prognosis [4, 5]. The search for biomarkers has been usually performed within the sepsis context. Though there have been described more than 170 potential biomarkers, still these have not reached the usefulness for prediction of severity and progression of infective processes [6, 7].

In contrast with the development in sepsis, biomarker searching in trauma patients is less advanced, being mostly restricted to traumatic brain injury (TBI). In this context, some of the reported markers show a good correlation with severity, brain damage and global outcome [8]. However, the use of these markers cannot be extended to other trauma types. In search of novel biomarkers to shed light upon pathophysiology venues, one should consider metabolomics. The use of metabolomics techniques allows the study of the complete set of low-molecular-weight intermediates (metabolites), which vary according to the pathologic state of the cell, tissue, organ, or organism and are context-dependent [9]. This approach offers a high number of potential biomarkers when combining the use of high-resolution chromatographic techniques with mass-spectrometry. The use of metabolomics in critical care management could help to a global understanding on metabolism after injury therefore leading to rationally derived therapeutic measures [6, 10].

Globally, despite there have been previous reports on the use of metabolomics in acute trauma care in the intensive care unit, these have been often restricted to the TBI context and/or non-chromatographic techniques (reviewed in [11]). To overcome these limitations, in the present work we have analyzed the metabolomic profiles of severe trauma patients admitted to an ICU, by comparing the early metabolomicprofiles of those patients who survived the trauma versus the ones belonging to non-survivors. The fact that a single metabolite may predict the fatal outcome after multiple injuries can be questioned. For this reason, we also evaluated if the total profile of metabolites recorded within 48 h after the trauma may define the risk of death within 14 days after admission to the intensive care unit. The results demonstrate the potential existence of early metabolomics markers of trauma-associated mortality.

## Methods

### Study design and participants

A prospective pilot study was designed with patients allocated to an ICU (Arnau de Vilanova University Hospital, Lleida, Spain) after a severe trauma. Protocol was supervised and approved by the Institutional Ethics Committee of the Arnau de Vilanova University Hospital. All participants (or their legal representatives) gave their written consent for the study. Inclusion criteria were all trauma-affected patients with severe status requiring ICU admission caused by a traumatic event in the prior 48 h, age > 16 years, with a follow-up period of 14 days in the ICU, in the period between 2011/10/01 and 2012/11/15. All patients whose samples were not obtained in the first 48 h were excluded.. Similarly, we excluded patients with acute kidney failure, defined as plasma creatinine higher than 1.5 times comparing to initial values or diuresis < 400 ml/24 h. Table 1 shows clinical features of included patients. None of the participants received artificial nutritional support in the first 48 h in ICU. Nutritional specificities were chosen on each patient individual basis, mainly according the possibility of enteral pathway and did not influence survival outcome.

**Table 1:** Demographic and clinical characteristics according to mortality

	ALL (*n* = 48)	SURVIVORS (*n* = 43)	NON SURVIVORS (*n* = 5)	*p* ^*^
Age (years)^a^	47,5 ± 19	45,7 ± 19	63,0 ± 10	*0,057*
Sex (Male) (%)	85,4	83,7	100,0	*0,438*
Traffic (%)	62,5	62,8	60,0	*0,706*
Severe Brain Injury (Glasgow score < = 8) (%)	37,5	34,9	60,0	*0,272*
Glasgow (score)	13 ± 3	13 ± 3	14 ± 3	*0,533*
MV (%)	37,5	30,2	100,0	*0,005*
NA (%)	33,3	30,2	60,0	*0,316*
PaO2/FiO2	305 ± 111	310 ± 112	265 ± 99	*0,429*
LOS (days)^b^	13(8–24)	16(8–28)	9(4–14)	*0,001*
APACHE II	11,6 ± 6	10,7 ± 6	19,0 ± 5	*0,010*
ISS	19,0 ± 9	18,9 ± 9	20,2 ± 7	*0,511*

Both APACHE II and ISS scores are directly proportional to severity of injury

*MV* Mechanical ventilation, *NA* noradrenaline perfusion, *PaO2/FiO2* Arterial Partial Pressure of Oxygen/Fraction of Inspired Oxygen, *LOS* Length of stay, *APACHE II* Acute Physiology and Chronic Health Evaluation II (ranges between 0 and 67), *ISS* Injury Severity Score (ranges between 0 and 75)

^*^p value after group comparison by χ^2^ or Mann-Whitney test for continuous variables

^a^mean ± standard deviation

^b^median (interquartile interval)

### Variables registered

Age, gender, length of ICU stay (LOS), Injury Severity Score (ISS) [12], Acute Physiology and Chronic Health Evaluation (APACHE II) [13]classification and mortality at 14 days were recorded. We also registered the severity of loss of respiratory function, measured as the lowest PaO_2_/FiO_2_ in the first 24 h of ICU admission, by using arterial gasometry, as well as mechanic ventilation (MV) need. Hemodynamic instability (shock) was diagnosed as the patient having a maintained (> 2 h) low systolic blood pressure level (< 90 mmHg) and/or requiring norepinephrine. Transfusion need was defined as those patients requiring more than one hemoconcentrate, and coagulopathy was defined as Quick score < 70% and/or thrombocyte count < 100,000/μL. All scales were validated by 2 independent evaluators.

### Sample processing

Blood was obtained in all cases by standard venipuncture or central venous catheterisation, between 24 and 48 h after the traumatic event in sodium citrate tubes. Immediately after sampling, diethylpentaacetic acid (1 mM) and butyl-hydroxy-toluene (10 μM) were added to the sample, in order to avoid artefactual oxidation. Plasma was obtained after centrifugation at 3000 x g, 4 °C for 10 min, and aliquoted into cryovials for immediate storage at − 80 °C. Samples were processed in a double-blind fashion, being aleatorized for extraction and injection. Metabolites from plasma were extracted as previously described [14]: samples were thawed at 4 °C, and 300 μl of cold methanol (containing 1 μg/ml of ^13^C-phenylalanine as internal standard) were added to 100 μl of plasma for deproteinization, followed by incubation at − 20 °C for 1 h and then, centrifuged at 12000 g for 3 min. The supernatants were recovered, evaporated using a Speed Vac (Thermo Fisher Scientific, Barcelona, Spain) and re-suspended in water 0.4% acetic acid/methanol (50/50).

### Metabolomic analyses

For the metabolomic study, an Agilent 1290 liquid chromatography system coupled to an ESI-Q-TOF MS/MS 6520 instrument (Agilent Technologies, Santa Clara, CA, US) was used. In all cases, 2 μL of extracted sample was applied onto a reversed-phase column (Zorbax SB-Aq 1.8 μm 2.1 × 50 mm; Agilent Technologies) equipped with a precolumn (Zorbax-SB-C8 Rapid Resolution Cartridge 2.1 × 30 mm 3.5 μm; Agilent Technologies) with a column temperature of 60 °C. The flow rate was 0.6 mL/min. Solvent A was composed of water containing 0.2% acetic acid and solvent B was composed of methanol 0.2% acetic acid. The gradient started at 2% B and increased to 98% B in 13 min and held at 98% B for 6 min. Post-time was established in 5 min.

Data were collected in positive electrospray mode time of flight operated in full-scan mode at 100–3000 m/z in an extended dynamic range (2 GHz), using N_2_ as the nebulizer gas (5 L/min, 350 °C). The capillary voltage was 3500 V with a scan rate of 1 scan/s. The ESI source used a separate nebulizer for the continuous, low-level (10 L/min) introduction of reference mass compounds: 121.050873, 922.009798 (positive ion mode) and 119.036320, 966.000725 (negative ion mode), which were used for continuous, online mass calibration. MassHunter Data Analysis Software (Agilent Technologies) was used to collect the results, and MassHunter Qualitative Analysis Software (Agilent Technologies) to obtain the molecular features of the samples, representing different, co-migrating ionic species of a given molecular entity using the Molecular Feature Extractor algorithm (Agilent Technologies), as described [14, 15]. Finally, MassHunter Mass Profiler Professional Software (Agilent Technologies) and Metaboanalyst platform [16] were used to perform a non-targeted metabolomic analysis of the extracted features. We selected samples with a minimum of 2 ions. Multiple charge states were not considered. Compounds from different samples were aligned using a retention time window of 0.1% ± 0.25 min and a mass window of 10.0 ppm ±2.0 mDa. We selected only those features in the 80-100th quartile of relative abundance and corrected for individual bias.

### Statistical analyses

The data are presented either as the mean ± standard deviation, the median (interquartile range) or as a percentage. Differences between survivor and non-survivors (NS) were evaluated with Chi-squared (for discrete variables) or with Mann-Whitney test by using the SPSS software ver 24 (IBM Corp, Armonk, NY, USA).

For metabolomic-derived variables, after log transformation and auto scaling of their abundances, multivariate statistics including Hierarchical Clustering, principal component analyses (PCA) and partial least square discriminant analyses (PLS-DA) were done using the Metaboanalyst platform [16]. The same platform was employed to evaluate differences between survivors and non surviving patients were evaluated by Student’s t test (*p* < 0.05), to generate receiver operating characteristic (ROC) curves, and to explore pathway impact (using hypergeometric test of over representation analyses). In the case of ROC curves, data were not log transformed nor autoscaled, being used the raw values of MS counts.

The identity of differential metabolites were annotated by searching their characteristics in the PCDL database from Agilent (Agilent Technologies, Barcelona, Spain), which uses retention times in a standardized chromatographic system as an orthogonal parameter to complement accurate mass data (accurate mass retention time approach) according to previously published works [16].

Mortality risk was calculated with binary regression logistic (both univariate and multivariate) by calculating *odds ratio* (OR) and confidence intervals (95%). Those markers with better discriminatory power according ROC curves were used. For the multivariate model we included those variables showing significance in the univariate approach, by using an stepwise selection type.

## Results

Table 1 shows clinical features of enclosed patients. APACHE II scores were significantly different between both groups (trauma surviving and non-surviving patients) analyzed. This agrees with previous data, showing that scores higher than 15 points have an overall mortality near 25%, and those scores higher than 25 a mortality of 50% [17] . When evaluating differences between trauma surviving and non-surviving patients there were almost significant differences in age, VM and LOS. All 5 non-surviving patients were victims of a high energy impact (traffic crash casualties or high level fall). In 4 cases, there was generalized hypoperfusion (haemorragic shock and hypoxia) and 1 case it was almost exclusively due to TBI. Of note, one of non-surviving patients had a very differential metabolomic signature (Addittional file 1: Figure S1). As it is shown, this patient presents very different relative abundance of all the metabolites and arouse as a potential outlier. When clinical parameters were check out we discovered that this patient suffered from amyotrophic lateral sclerosis so we discarded the corresponding metabolomics profile for further multivariate analyses, with TBI as predominant mechanism. No patients with sepsis were present in our cohort.

**Fig. 1:**
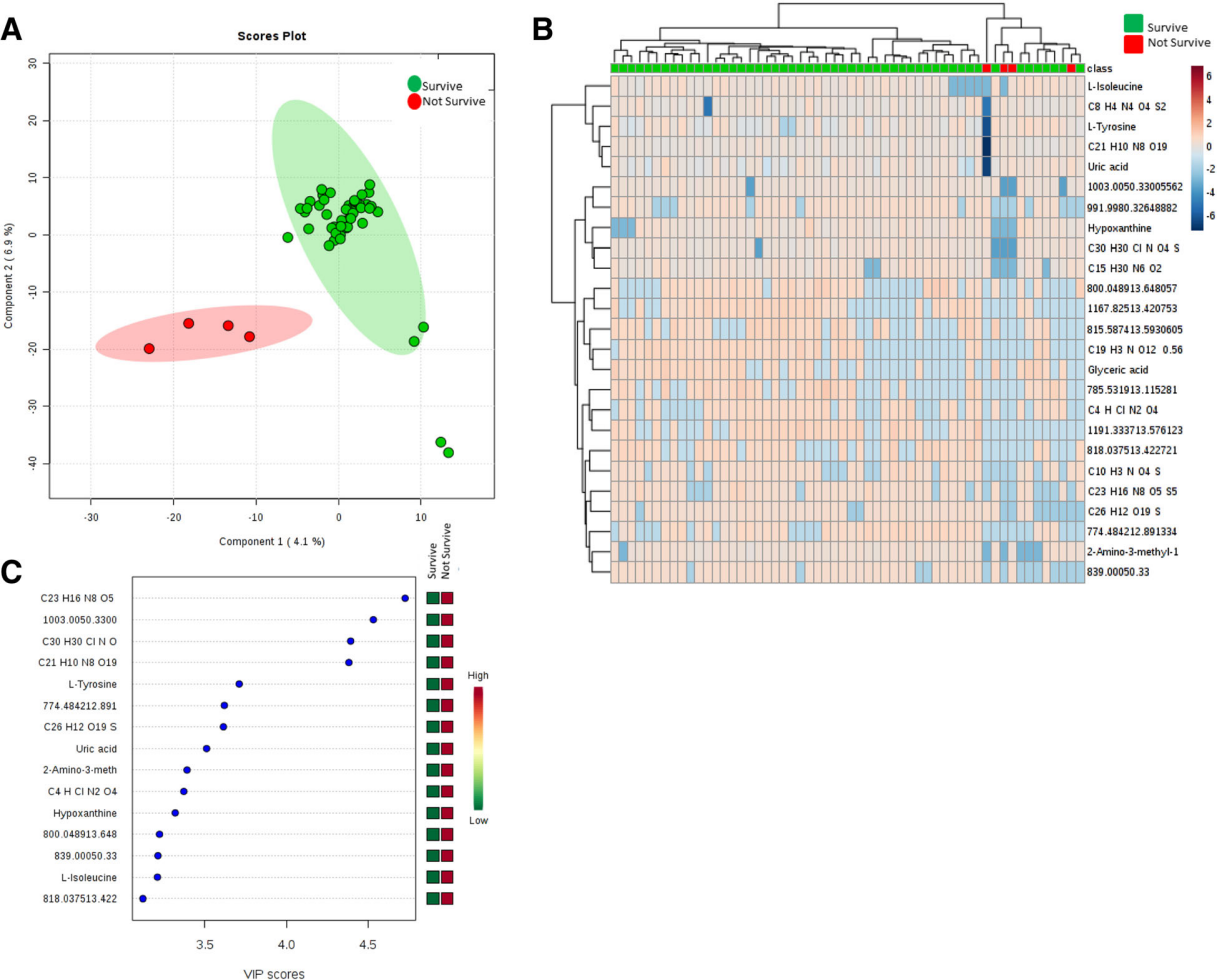
Patients non surviving severe trauma exhibit a metabolomic signature. **a** PLS-DA analyses shows that metabolome is able to discriminate between those patients who survive and those patients who do not survive, with those metabolites contributing to separation shown in the VIP score graph (**c**). **b** Heatmap hierarchical clustering analyses using the 25 metabolites with the lowest *p* value (T-Student test) indicates that there is not a perfect clusterization between groups. PLS-DA cross-validation details (4 components): Accuracy: 0.92, R2: 0.97, Q2: -0.189. The negative value of Q2 means that the model is not all predictive or is overfitted, probably because the low number of not surviving patients

By employing metabolomics we were able to detect 2084 molecular features in plasma (see Declaration section for data availability). As shown in Fig. 1, plasma from non-surviving patients exhibited a specific metabolomic signature in plasma (Fig. 1a) by both PLS-DA and hierarchical clustering analyses. The most important metabolites defining this signature are shown in Fig. 1c. Among them, we were able to identify L-Tyrosine, Uric acid, 2-Amino-3-methyl-1-butanol, hypoxanthine and L-Isoleucine. As shown in Fig. 1b the discrimination between groups using hierarchical clustering algorithm with 25 molecules with the lowest *p* value (T-Student Test) had a non-optimal performance, with some surviving patients clustered together with non-surviving patients.

**Fig. 2:**
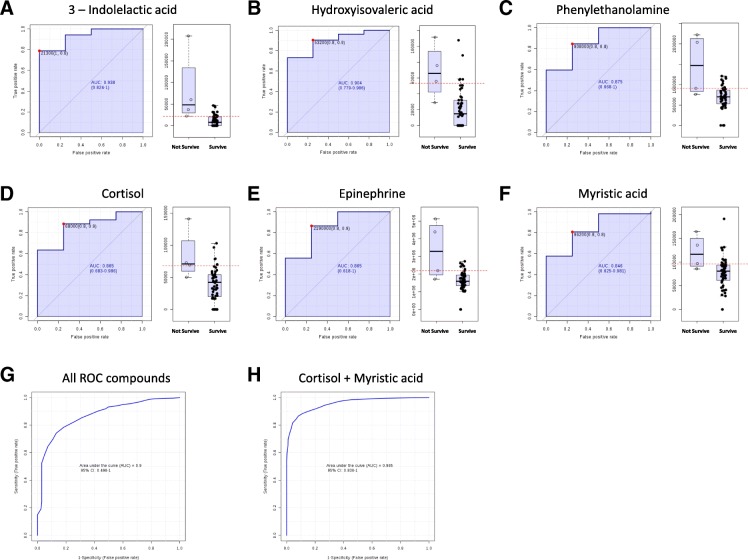
ROC curves of those metabolites with a potential identity and higher AUC values (**a-f**). **g** ROC curve using the values of all the metabolites from (**a**) to (**f)**. **h** ROC curve using Cortisol and Myristic acid

When applying univariate analyses (Student T-Test) 84 significantly different molecules (p < 0.05, 10 upregulated in surviving patients, 74 in non-surviving patients) appeared (Table 2 for annotated molecules and Additional file 1: Table S1 for full details). In order to define the capacity of specific metabolites as potential biomarkers, ROC curves were also performed using those metabolites in 80-100th percentile of abundance. Among the ROC curves with higher area under the curve (AUC), we selected those annotated with a potential identity basing on exact mass and retention time (Fig. 2). All these selected metabolites (3-Indolelactic acid (AUC: 0.938), hydroxyisovaleric acid (AUC: 0.904), phenylethanolamine (AUC: 0.875), cortisol (AUC: 0.865), epinephrine (AUC: 0.865), and myristic acid (AUC: 0.846)) were increased in plasma of non-surviving patients. To further evaluate the capacity of these metabolites we combined their values. Figure 2g shows that combining all these selected metabolites we achieved an AUC = 0.9 and, interestingly, using only the values of cortisol and myristic acid the AUC values reach 0.965. The discrimination capacity was lower than those of APACHE II or ISS (Table 1). Further, no significant correlation was found between these clinical scores and chosen metabolites for further analyses (cortisol, myristic acid), as shown in Table 3. Nonetheless, as shown in Table 4, these metabolomic biomarkers, as well as APACHE II score, were considered risk factors in univariate mortality risk. Noteworthy, myristic acid level was the only independent risk factor in multivariate models of mortality.

**Table 2:** Annotated differential metabolites according to mortality

Name of potential metabolite^a^	t.stat	*P* value^*^	False Discovery Rate corrected p
3-Indolelactic acid	5.6738	5.66E-07	0.0010485
Epinephrine	5.0119	6.15E-06	0.005697
Phenylethanolamine	4.6647	2.07E-05	0.009615
Hydroxyisovaleric acid	3.5859	0.0007228	0.13401
Cortisol	3.2222	0.0021581	0.23536
L-Tryptophan	3.0729	0.0033195	0.30045
Myristic acid	2.4577	0.017226	0.7234
Pyridoxal	2.4207	0.018882	0.74483
Bilirubin	2.3772	0.021017	0.78289
Erythrono-1,4-lactone	2.0616	0.044076	0.88671
Elaidic Acid	2.0106	0.049371	0.88671

^*^after Student t test

^a^Potential identity based on isotope distribution, exact mass and retention time similarity with the PCDL from Agilent

**Table 3:** Correlation between potential biomarkers of mortality (*n* = 48)

	APACHE II	ISS	CORTISOL	MYRISTIC ACID
APACHE II	–			
ISS	0,488*	–		
CORTISOL	0,245	−0,172	–	
MYRISTIC ACID	0,215	−0,067	0,292*	–

Values shown are Spearman correlation coefficients with * *p* < 0,05

*APACHE II* Acute Physiology and Chronic Health Evaluation*, ISS* Injury Severity Score

**Table 4:** Mortality Risk Model (binary logistic regression)

Variable	UNIVARIANT OR (CI 95%)	MULTIVARIANT OR (CI 95%)
APACHE II	1,2 (1,0 – 1,5)	NS
CORTISOL	1,6 (1,0 – 8,4)	NS
MYRISTIC ACID	2,1 (1,1 – 3,9)	2,1 (1,1 – 3,9)

*OR* Odds Ratio, *CI* Confidence interval, *APACHE II* Acute Physiology and Chronic Health Evaluation

**Fig. 3:**
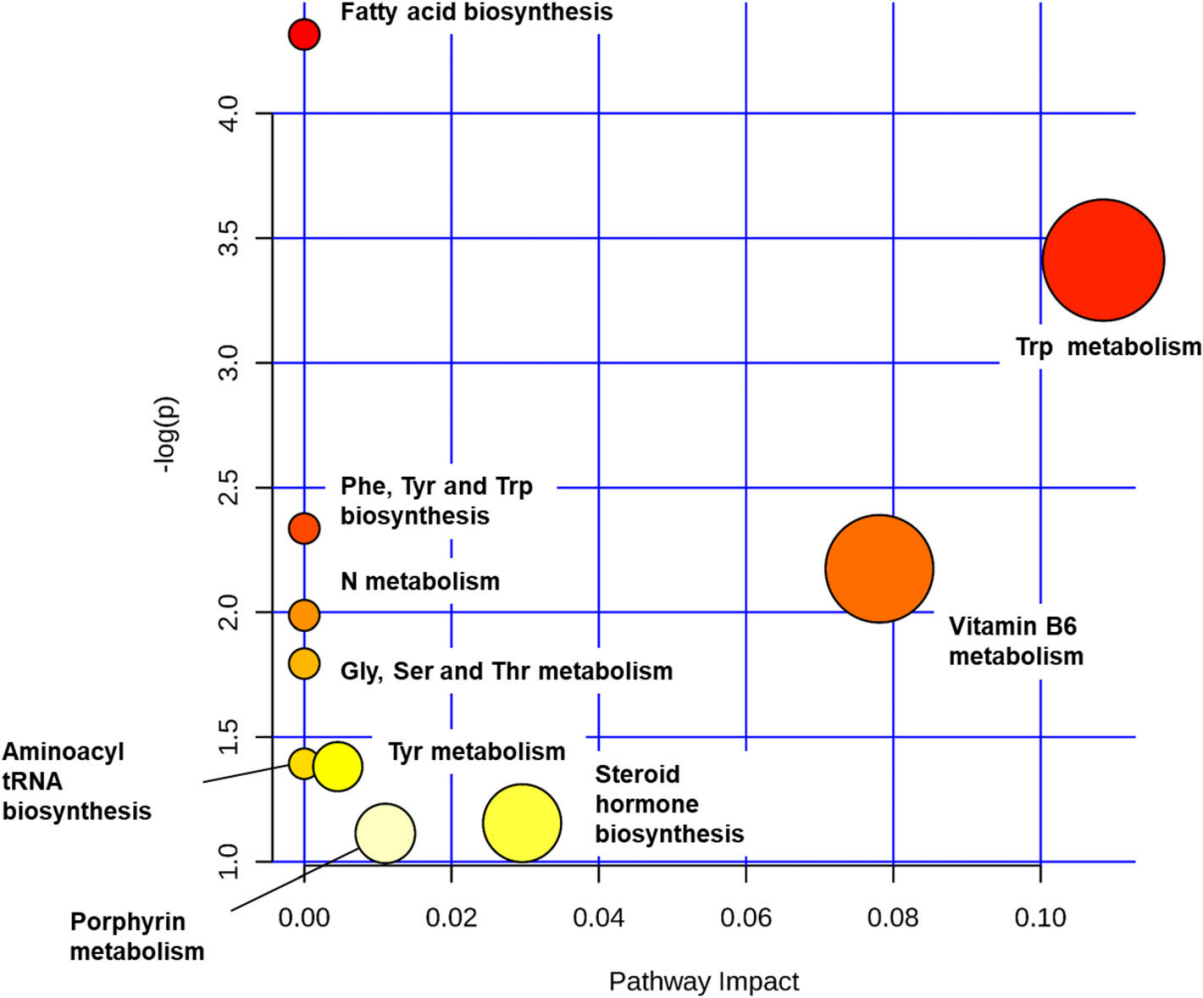
Pathway impact of differential metabolites according survival. The 11 differential metabolites annotated (Indolelactic acid, Epinephrine, Phenylethanolamine, 2-Hydroxyisovaleric acid,Cortisol, L-Tryptophan, Myristic acid, Pyridoxal, Bilirubin, Erythrono-1,4-lactone, Elaidic Acid) were searched against KEGG pathway library of *H Sapiens* using an hypergeometric test for over representation analyses. *P* value is encoded in intensity of red color of circles (representing pathways) and size of the circles represent the diversity of the pathway, based on the number of metabolites contained in the pathway

Pathway analyses reveal the 11 annotated metabolites clustered (*p* = 0.11) along several amino-acid related pathways (Fig. 3), including fatty acid biosynthesis, and tryptophan and tyrosine metabolism.

## Discussion

The presented results highlight the usefulness of metabolomic approaches in the characterization of pathophysiological events after severe trauma, as suggested by previous reviews [11]. To the best of our knowledge, this is one the first reports using untargeted liquid chromatography coupled to mass spectrometry (LC-MS) in plasma for a non-favorable outcome in a severe trauma population. Previous results, using nuclear magnetic resonance (NMR) have shown that there are clear age and gender-dependent differences in energy metabolism and oxidative stress response in case-control approaches [18]. In a similar case-control study, Brodie et al. showed metabolomics changes in oxidative stress, nucleotide synthesis and muscle catabolism [10], but in this case, they employed targeted LC-MS. However, the study was not addressed for mortality. In a similar study, comprising 95 severely injured patients, targeted metabolomics for lactate and succinate revealed its potential as markers for negative outcome [19]. Similarly, an NMR-based study in the context of combat injuries revealed also succinate as marker of mortality, in addition to malonate [20]. However, the number of metabolites potentially detectable are lower than in our study, for technical limitations. Other studies have focused on the use of plasma metabolomics in TBI management [21, 22], even using cerebrospinal fluid [23] or urine [24] as the sample of reference. Similarly, in a case-control approach, other researchers indicate that a low number of metabolites, one of them stearic acid, allowed for differentiation of TBI from controls, and even helped to classify TBI patients according severity [25].

In our cohort, six annotated metabolites defined survival chances with a very high accuracy. The finding of cortisol agrees with previous reported data [26]. However, these contrast with other reports [27], where vasopressor-dependent critical illness individuals do not show the same behavior. Differences in gender, organ dysfunction and other clinical features could explain this difference. Pathophysiologically, this could be linked to poor glycemic control attributable to high cortisol levels [28]. Interestingly, cortisol is one of the physiological mediators of hyperaminoacidemia [29] and increased branched chain aminoacid levels [30] found in the postinjury phase by previous metabolomic approaches [10]. Of note, epinephrine also predicted low chances of survival. This result agree with previous series, where high catecholamine values were associated to adverse cardiac events [31], or to higher mortality, via endothelial damage and glycocalix degradation, as well as hyperfibrinolysis [32]. The increase of the trace amine phenylethanolamine can be ascribed to the same phenomenon, as it is considered a by-product of catecholamine synthesis [33, 34]. Nonetheless, no previous report on its usefulness as mortality biomarker was present.

3-indolelactic acid is a metabolite of tryptophan degradation [35]. No previous results related this metabolite to non-favorable outcome in trauma. Recent metabolomics reports indicate that this marker is decreased in plasma of patients with cachexia [36]. Nonetheless, previous results with experimental models of cardiac arrest, reveal that tryptophan catabolism was directly related to non-survival outcome [37]. Similarly, high catabolism of tryptophan has been also associated to mortality in bacteremia [38]. Levels of quinolinic acid in cerebrospinal fluid, other of the tryptophan metabolites, are strongly related to low survival chances in TBI. The fact that tryptophan metabolism is increased in trauma patients, even after intravenous aminoacid infusion, is recognized since long time ago [39].

Concerning myristic acid, this long chain fatty acid has marked effect in hepatic cells [40], leading to cellular stress and steatosis. Independent results have revealed that it also confers cardiovascular risk [41]. Myristic acid in blood may be the result of increased lypolisis in adipose tissue, one of the physiological effects of cortisol and epinephrine. However, why only this fatty acid, and not other shorter or longer chain fatty acid correlate with mortality is not known. In cellular terms, myristolation is a key event [42] in immune response, especially in innate immune response, lymphopoiesis for T cells, and the formation of the immunological synapse. Of note, recent evidences link trauma to immune disarrangements, through the release of the so-called damage-associated molecular patterns (DAMPs). DAMPs are considered danger signals which, paradoxically, lead to propagation of injuries to remote tissues, contributing to multiple organ failure and even death (reviewed in [43]). Whether the changes in myristic acid are associated to DAMPs buildup, release or signaling is yet unknown. Other metabolomic works discovered octanoic and decanoic fatty acids, structurally related to myristic, as potential biomarkers of severity in TBI patients [44]. Previous works, employing NMR also uncovered lipids (in their case monounsaturated fatty acids, triacylglycerol and phospholipids) as predictors for survival in the context of trauma [45].

Myristic acid belongs to long chain free fatty acids (FFAs). The relationship of FFAs with a negative outcome are not new, as their concentrations in plasma are directly associated to trauma severity scores [46]. Further, they are increased after bone fracture [47]. Further, non-trauma related intensive-care conditions such as burn or acute pancreatitis injuries also lead to increased concentrations of FFAs [48–50]. FFAs could trigger myocardial injury through TLR4 activation [51], and they have been directly related with cardiovascular mortality [52]. In the context of trauma, they inhibit the production of an anti-inflammatory interleukin, IL-10 [53] In addition, they directly induce necrosis [54] through membrane modifications, which could also include increased susceptibility to hemolysis, a well-known condition with negative consequences [55]. Noteworthy, recent data show that differences in albumin FFA-binding properties could explain different capacities of albumin infusions to modulate cell damage in ICU environment [56]. Of note, previous works from our work revealed an impact of FFAs as determinants of lifespan in comparative physiology approaches [57], highlighting the potential influence of circulating FFAs in insulin signaling and other relevant physiological processes [58].

Our study shows some limitations. Regarding the selection of possible biomarkers based on the Student t-test can be questioned, because its use to study differences between groups different in size and variance is prone to errors. This is evident in the box and whisker plots of each of the proposed biomarkers, where some of the highest readings in the surviving group are very similar to the results of the non-survivors. Nonetheless, the ROC curves of the selected biomarkers showed high diagnostic accuracy. PLS-DA model statistics for non-survival could show some overfitting, due to relative low number of samples. In our cohort, mortality was low (ca 10%). In addition, almost no mortality was evident by TBI (which shows a high mortality in the first 96 h after trauma), and many non-surviving patients overcoming the first 48 h could show multiorgan failure, with probably a marked influence in metabolome. Further, those patients with higher severity could require fluid therapy, thereby also conditioning metabolic profile. Similarly, differences in metabolomics profiles due to fasting-fed state before trauma could be also a limitation, as suggested by data in preclinical models [59]. Additionally, age differences, mechanisms of trauma, sex, and body mass index could also impinge changes in metabolome. Of note, this study does not offer a confirmatory cohort, which may be useful for enhancing robustness of findings. Further meta-analyses resulting from exploration with larger datasets/higher mortality rates/highly stratified injury severities are also warranted. However, even with these limitations, similarly sized studies have been reported on the discovery of metabolome-based biomarkers of mortality [20].

We recognize that finding similar candidates within other studies which may show some underpowered sampling does not completely validate our findings. However, these reported studies have been obtained with different analytical techniques, across different geographic environments, thereby suggesting a common pathophysiological foundations. In this respect, the routine use of LC-MS based metabolomics is still not achievable in most hospitals, due to cost-benefit constraints. Nonetheless, LC-MS based metabolomics are helpful for proposal of novel potential biomarkers, enhancing patient’s stratification. Further, its application could pave the way for discovery of pathophysiologically relevant pathways, hereby leading to rationally designed and personalized therapeutics which should enhance the prognosis of these patients.

## Conclusions

Overall, revealed metabolic pathways disclose a catecholamine and cortisol stress response with a potential role of fatty acid metabolism and degradation of specific amino acids. Further, the fact that multivariate and ROC analyses show a better behavior than consolidated clinical markers could be helpful in the search for measures enhancing prognosis in these patients.

## Additional file

Additional file 1: **Table S1.** Differential metabolites according mortality (full list). **Figure S1.** Clinical specificity for metabolomics signature. A. PLS-DA analyses shows that metabolome is able to discriminate between those patients who survive and those patients who do not survive. Although the metabolomic profile of survived patients is homogenous, one of the non-surviving patients had a specific metabolomic profile (black arrow). B. Heatmap hierarchical clustering analyses using the 25 metabolites with the lowest *p* value (T-Student test) confirms that one patient has a specific metabolomic profile (black arrow). PLS-DA cross-validation details (4 components): Accuracy: 0.91, R2: 0.96, Q2: -0.141. The negative value of Q2 means that the model is not all predictive or is overfitted, probably because the low number of not surviving patients. (DOCX 239 kb)

## Abbreviations

APACHE II: Acute Physiology and Chronic Health Evaluation
AUC: Area Under the Curve
DAMPs: Damage-Associated Molecular Patterns
ESI: Electrospray Ionization
FFAs: Free Fatty Acids
FiO: Fraction of Inspired Oxygen
ICU: Intensive Care Unit
ISS: Injury Severity Score
KEGG: Kyoto Encyclopedia of Genes and Genomes
LC-MS: Liquid Chromatography Coupled to Mass Spectrometry
LOS: Length of ICU Stay
MS: Mass-spectrometry
MV: Mechanic Ventilation
NMR: Nuclear Magnetic Resonance
OR: Odds Ratio
PaO2: Arterial Partial Pressure of Oxygen
PCA: Principal Component Analyses
PLS-DA: Partial Least Square Discriminant Analyses (PLS-DA)
Q-TOF: Quadrupole Time-Of-Flight
RISC: Revised Injury Severity Classification
ROC: Receiver Operating Curve
SAPS: Simplified Acute Physiologic Score
TBI: Traumatic Brain Injury
TRAM: Trauma Risk Adjustment Model
TRISS: Trauma and Injury Severity Score
