# Supplementary material for: A prospective pilot study using metabolomics discloses specific fatty acid, catecholamine and tryptophan metabolic pathways as possible predictors for a negative outcome after severe trauma
Source: Scand J Trauma Resusc Emerg Med. 2019 May 22;27:56. doi: 10.1186/s13049-019-0631-5 (PMC6530007; doi:10.1186/s13049-019-0631-5)
Supplement: Supplementary file 1 — Table S1. Differential metabolites according mortality (full list). Figure S1. Clinical specificity for metabolomics signature. A. PLS-DA analyses shows that metabolome is able to discriminate between those patients who survive and those patients who do not survive. Although the metabolomic profile of survived patients is homogenous, one of the non-surviving patients had a specific metabolomic profile (black arrow). B. Heatmap hierarchical clustering analyses using the 25 metabolites with the lowest p value (T-Student test) confirms that one patient has a specific metabolomic profile (black arrow). PLS-DA cross-validation details (4 components): Accuracy: 0.91, R2: 0.96, Q2: -0.141. The negative value of Q2 means that the model is not all predictive or is overfitted, probably because the low number of not surviving patients. (DOCX 239 kb) [file 13049_2019_631_MOESM1_ESM.docx]

Additional file 1: Table S1. Differential metabolites according mortality (full list)

| Metabolite^1^ | t.stat | p.value^2^ | False Discovery rate corrected p value |
| --- | --- | --- | --- |
| 3-Indolelactic acid | 5.6738 | 5.66E-07 | 0.0010485 |
| Epinephrine | 5.0119 | 6.15E-06 | 0.005697 |
| C8 H6 | 4.719 | 1.72E-05 | 0.009615 |
| Phenylethanolamine | 4.6647 | 2.07E-05 | 0.009615 |
| C4 H5 N3 S3 | 4.1532 | 0.00011741 | 0.043534 |
| C31 H42 N4 O S3 | 4.0165 | 0.00018397 | 0.056846 |
| C6 H4 | 3.7724 | 0.00040317 | 0.10678 |
| C37 H30 Cl N5 O S | 3.6192 | 0.00065192 | 0.13401 |
| Unknown | -3.6088 | 0.00067331 | 0.13401 |
| Hydroxyisovaleric acid | 3.5859 | 0.0007228 | 0.13401 |
| C38 H60 Cl N9 | 3.5326 | 0.0008516 | 0.14353 |
| C9 H8 O2 | 3.4011 | 0.0012696 | 0.19616 |
| C24 H45 N7 O2 | 3.3137 | 0.0016484 | 0.23509 |
| C42 H87 N3 O S3 | 3.2631 | 0.0019142 | 0.23536 |
| Unknown | 3.2453 | 0.0020168 | 0.23536 |
| C39 H32 Cl N3 O4 | 3.2403 | 0.0020464 | 0.23536 |
| Cortisol | 3.2222 | 0.0021581 | 0.23536 |
| C11 H9 N O2 | 3.0841 | 0.0032153 | 0.30045 |
| C19 H41 N7 O2 S | 3.0802 | 0.0032508 | 0.30045 |
| L-Tryptophan | 3.0729 | 0.0033195 | 0.30045 |
| C45 H72 Cl N3 O | 3.0641 | 0.0034031 | 0.30045 |
| C39 H34 N2 | 2.9814 | 0.0042974 | 0.33204 |
| C44 H79 N O5 S | 2.9791 | 0.0043245 | 0.33204 |
| C13 H16 N2 O4 | 2.9758 | 0.0043649 | 0.33204 |
| C33 H54 O5 S2 | 2.9667 | 0.0044774 | 0.33204 |
| Unknown | 2.9292 | 0.004969 | 0.35433 |
| Unknown | 2.8793 | 0.0057016 | 0.39151 |
| Unknown | 2.8639 | 0.0059481 | 0.39385 |
| C15 H14 N4 | 2.8023 | 0.0070304 | 0.43336 |
| C18 H25 N5 O S3 | 2.7952 | 0.0071668 | 0.43336 |
| C33 H34 N4 O6 | 2.7911 | 0.007246 | 0.43336 |
| C30 H30 Cl N O4 S | -2.7678 | 0.0077139 | 0.44693 |
| C30 H62 N2 O3 S2 | 2.7367 | 0.0083838 | 0.47102 |
| C26 H12 O19 S | -2.6505 | 0.010523 | 0.57381 |
| C26 H41 N5 | 2.6064 | 0.011802 | 0.62516 |
| C7 H15 N O3 | 2.5671 | 0.013059 | 0.66674 |
| Unknown | 2.5574 | 0.013386 | 0.66674 |
| Unknown | -2.5493 | 0.013666 | 0.66674 |
| Unknown | 2.4975 | 0.015589 | 0.72259 |
| C33 H36 N4 O6 | 2.4974 | 0.01559 | 0.72259 |
| Unknown | 2.4815 | 0.016229 | 0.7234 |
| Myristic acid | 2.4577 | 0.017226 | 0.7234 |
| Unknown | 2.4525 | 0.017448 | 0.7234 |
| C36 H54 O S | 2.4517 | 0.017484 | 0.7234 |
| C22 H35 N O2 | 2.4472 | 0.01768 | 0.7234 |
| C16 H20 N6 O3 S | 2.4412 | 0.017948 | 0.7234 |
| Pyridoxal | 2.4207 | 0.018882 | 0.74483 |
| Unknown | 2.3924 | 0.020248 | 0.78207 |
| Bilirubin | 2.3772 | 0.021017 | 0.78289 |
| Unknown | -2.3576 | 0.022047 | 0.78289 |
| C15 H37 N7 9.0476465 | 2.3523 | 0.022335 | 0.78289 |
| Unknown | 2.3495 | 0.022485 | 0.78289 |
| C31 H54 S2 | 2.3477 | 0.022582 | 0.78289 |
| Unknown | 2.3437 | 0.022803 | 0.78289 |
| C33 H44 O6 S2 | 2.315 | 0.024441 | 0.81724 |
| 421.756613.325806 | 2.3058 | 0.024992 | 0.81724 |
| C15 H28 N2 S2 | 2.3035 | 0.025125 | 0.81724 |
| C40 H82 N4 S2 | 2.2703 | 0.027202 | 0.86952 |
| C13 H10 N4 | 2.2426 | 0.02905 | 0.88671 |
| C33 H16 N8 O3 S5 | 2.2319 | 0.029789 | 0.88671 |
| C9 H18 N2 S | 2.2284 | 0.030035 | 0.88671 |
| C4 H Cl N2 O4 | -2.2223 | 0.030473 | 0.88671 |
| 773.603213.69954 | 2.222 | 0.030493 | 0.88671 |
| C7 H16 N6 S | 2.1926 | 0.032662 | 0.88671 |
| C40 H79 N O5 S2 | 2.187 | 0.033096 | 0.88671 |
| 1168.832813.40572 | 2.186 | 0.033172 | 0.88671 |
| 812.661113.753789 | 2.1824 | 0.03345 | 0.88671 |
| C31 H40 N4 | 2.149 | 0.036135 | 0.88671 |
| C39 H65 Cl O2 | 2.147 | 0.036305 | 0.88671 |
| C5 H10 Cl N O | 2.1437 | 0.036577 | 0.88671 |
| 835.99360.32962006 | -2.1133 | 0.039212 | 0.88671 |
| C45 H81 N S | 2.0956 | 0.040821 | 0.88671 |
| C17 H36 N2 O4 | 2.0924 | 0.04112 | 0.88671 |
| Unknown | 2.0868 | 0.041645 | 0.88671 |
| Unknown | -2.077 | 0.04257 | 0.88671 |
| Erythrono-1,4-lactone | 2.0616 | 0.044076 | 0.88671 |
| Unknown | 2.057 | 0.044533 | 0.88671 |
| Unknown | -2.0438 | 0.045859 | 0.88671 |
| Unknown | 2.0387 | 0.046391 | 0.88671 |
| C11 H26 N6 | 2.0351 | 0.046766 | 0.88671 |
| C16 H7 N O12 S | 2.0237 | 0.047958 | 0.88671 |
| Unknown | -2.0211 | 0.048242 | 0.88671 |
| C19 H19 N11 | -2.0149 | 0.048906 | 0.88671 |
| Elaidic Acid | 2.0106 | 0.049371 | 0.88671 |

^1^ Potential ID based on isotope distribution, exact mass and retention time similarity;^2^ after Student t test

**Adittional file 1: FIGURE S1**


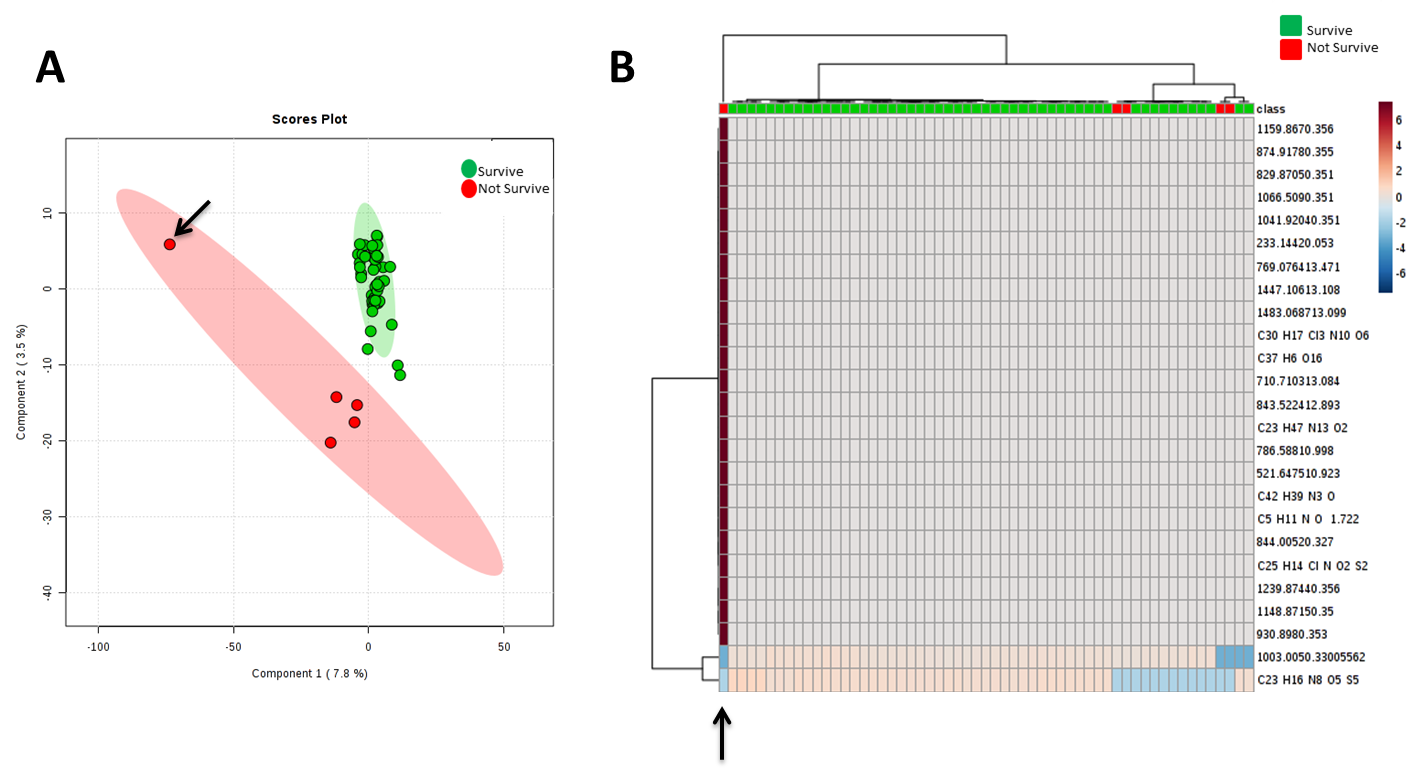


Additional file 1: figure S1. Clinical specificity for metabolomics signature. A. PLS-DA analyses shows that metabolome is able to discriminate between those patients who survive and those patients who do not survive. Although the metabolomic profile of survived patients is homogenous, one of the non suriving patients had a specific metabolomic profile (black arrow). B. Heatmap hierarchical clustering analyses using the 25 metabolites with the lowest p value (T-Student test) confirms that one patient has a specific metabolomic profile (black arrow). PLS-DA cross-validation details (4 components): Accuracy: 0.91, R2: 0.96, Q2: -0.141. The negative value of Q2 means that the model is not all predictive or is overfitted, probably because the low number of not surviving patients.
